# Cerebellar Vermis and Midbrain Hypoplasia Upon Conditional Deletion of *Chd7* from the Embryonic Mid-Hindbrain Region

**DOI:** 10.3389/fnana.2017.00086

**Published:** 2017-10-04

**Authors:** Alex P. A. Donovan, Tian Yu, Jacob Ellegood, Kimberley L. H. Riegman, Christa de Geus, Conny van Ravenswaaij-Arts, Cathy Fernandes, Jason P. Lerch, M. Albert Basson

**Affiliations:** ^1^Centre for Craniofacial and Regenerative Biology, King’s College London, London, United Kingdom; ^2^Department of Medical Biophysics, University of Toronto, Mouse Imaging Centre, Hospital for Sick Children, Toronto, ON, Canada; ^3^Department of Genetics, University of Groningen, University Medical Center Groningen, Groningen, Netherlands; ^4^MRC Social, Genetic & Developmental Psychiatry Centre, Institute of Psychiatry, Psychology & Neuroscience, King’s College London, London, United Kingdom; ^5^MRC Centre for Neurodevelopmental Disorders, King’s College London, London, United Kingdom; ^6^Department of Medical Biophysics, University of Toronto, Toronto, ON, Canada

**Keywords:** CHD7, mid-hindbrain, cerebellum, vermis, hypoplasia

## Abstract

Reduced fibroblast growth factor (FGF) signaling from the mid-hindbrain or isthmus organizer (IsO) during early embryonic development results in hypoplasia of the midbrain and cerebellar vermis. We previously reported evidence for reduced *Fgf8* expression and FGF signaling in the mid-hindbrain region of embryos heterozygous for *Chd7*, the gene mutated in CHARGE (Coloboma, Heart defects, choanal Atresia, Retarded growth and development, Genitourinary anomalies and Ear defects) syndrome. However, *Chd7*^+/−^ animals only exhibit mild cerebellar vermis anomalies. As homozygous deletion of *Chd7* is embryonic lethal, we conditionally deleted *Chd7* from the early embryonic mid-hindbrain region to identify the function of CHD7 in mid-hindbrain development. Using a combination of high resolution structural MRI and histology, we report striking midbrain and cerebellar vermis hypoplasia in the homozygous conditional mutants. We show that cerebellar vermis hypoplasia is associated with reduced embryonic *Fgf8* expression and an expanded roof plate in rhombomere 1 (r1). These findings identify an essential role for *Chd7* in regulating mid-hindbrain development via *Fgf8*.

## Introduction

The mammalian cerebellum consists of a medial vermis, flanked by two hemispheres. Genetic lineage tracing studies in the mouse have shown that the cerebellar vermis is derived from a small group of progenitor cells located in the most anterior part of rhombomere 1 (r1; Sgaier et al., 2005). The specification, maintenance and/or expansion of these progenitors is regulated by the fibroblast growth factor (FGF) signaling pathway (Joyner et al., 2000; Chi et al., 2003; Sato et al., 2004; Sgaier et al., 2005; Basson et al., 2008). FGF ligands, the most prominent being FGF8, are produced by the isthmus organizer (IsO), the secondary signaling center that forms at the boundary between the embryonic mesencephalon (mes) and r1 (reviewed by Basson and Wingate, 2013). We have previously shown that the level of FGF signaling from the IsO has to be tightly controlled. Whereas increased signaling results in an expanded vermis (Yu et al., 2011), reduced FGF signaling leads to cerebellar vermis hypoplasia (Basson et al., 2008).

We previously identified the chromatin remodeling factor CHD7 as an essential upstream regulator of *Fgf8* gene expression (Yu et al., 2013), consistent with its function in fine-tuning developmental gene expression (Schnetz et al., 2010). Indeed, reduced *Chd7* expression in *Chd7*^+/−^ mouse embryos on C57BL/6J and C57BL/6J × DBA/2J backgrounds was associated with reduced *Fgf8* expression (Yu et al., 2013). Intriguingly, these *Chd7* heterozygous mice did not exhibit overt cerebellar vermis hypoplasia or aplasia (Yu et al., 2013). Striking cerebellar vermis hypoplasia became evident on an *Fgf8*^+/−^ background, indicative of a strong genetic interaction between *Chd7* and *Fgf8* loss of function alleles (Yu et al., 2013; Basson, 2014). An analysis of *Chd7*^−/−^ embryos found a strong reduction in *Fgf8* expression and loss of r1 identity (Yu et al., 2013). However, these homozygous *Chd7* embryos die by ~E11 (Hurd et al., 2007; Randall et al., 2009), precluding an analysis of cerebellar development and structure after this stage in embryos lacking *Chd7* expression during mid-hindbrain development.

Here, we deleted *Chd7* from the mes/r1 region in mouse embryos using a conditional gene targeting approach and asked whether *Chd7* deletion from the mes/r1 region, which includes the IsO, was sufficient to cause cerebellar vermis and midbrain hypoplasia.

## Materials and Methods

### Mice

The *En1*^*cre*/+^ and *Chd7*^*flox*^ mouse lines have been described (Kimmel et al., 2000; Jones et al., 2015). Both these alleles were backcrossed onto the C57BL/6J background for at least three generations and genotyped according the original publications. Conditional mes/r1-specific *Chd7* conditional knockout *En1*^*cre*/+^;*Chd7*^*flox*/*flox*^ (*En1*^*cre*/+^;*Chd7*^*f*/*f*^) mice were produced by *En1*^*cre*/+^;*Chd7*^*flox*/+^ × *Chd7*^*flox*/*flox*^ crosses. Mice were bred and maintained in the Biological Services Unit at Guy’s Campus or the Institute of Psychiatry, Psychology and Neuroscience, King’s College London. All procedures involving animals were approved by the local ethical review panel of King’s College London, and the U.K. Home Office Animals Scientific Procedures Act 1986. The work was carried out under licenses (PPL70/6694 and PPL70/7184) and all efforts were made to minimize animal suffering and to reduce the number of animals used.

### Histology

Brains or embryos were dissected in phosphate buffered saline (PBS), fixed overnight in 4% paraformaldehyde (PFA) at 4°C, dehydrated and embedded in paraffin wax. Serial, sagittal sections were cut at 10 μm and left to dry overnight at 42°C. Sections were stained with Cresyl Violet or processed for immunohistochemistry as described (Whittaker et al., 2017). The following primary antibodies were used: anti-tyrosine hydroxylase (Abcam, ab112; 1:200) and anti-Neurogranin (Millipore, AB5620; 1:500). Primary antibodies were detected using Alexa fluor-conjugated secondary antibodies (Invitrogen; 1:200) or biotinylated secondary antibodies (Dako, E0466; 1:200) with the Vectastain ABC Kit (Vector Laboratories) and visualized using 0.03% diaminobenzidine (DAB; Sigma).

### *In Situ* Hybridization

*In situ* hybridization was performed using standard methods (Basson et al., 2008; Yaguchi et al., 2009). The *Fgf8* and *Etv5 in situ* hybridization probes were reported by Yaguchi et al. (2009) and the *Chd7* exon 3 probe by Whittaker et al. (2017).

### Structural MRI

A total of 45 mice were examined in this study. The mouse numbers used were: 13 *Chd7*^*flox*/*flox*^, 12 *En1*^*cre*/+^, 10 *En1*^*cre*/+^;*Chd7*^*flox*/+^ and 10 *En1*^*cre*/+^;*Chd7*^*flox*/*flox*^. All mice were adults (P80–P100). Mice were terminally anesthetized and intracardially perfused with 30 mL of 0.1 M PBS containing 10 U/mL heparin and 2 mM ProHance (Bracco Diagnostics Inc.), a Gadolinium contrast agent followed by 30 mL of 4% PFA containing 2 mM ProHance (Spring et al., 2007; Cahill et al., 2012). Perfusions were performed at a rate of approximately 60 mL/h. After perfusion, mice were decapitated. The brain and remaining skull structures were incubated in 4% PFA + 2 mM ProHance overnight at 4°C then transferred to 0.1 M PBS containing 2 mM ProHance and 0.02% sodium azide for at least 1 month days prior to MRI scanning (De Guzman et al., 2016). A multi-channel 7.0 Tesla MRI scanner (Agilent Inc., Palo Alto, CA, USA) was used to image the brains within skulls. Sixteen custom-built solenoid coils were used to image the brains in parallel (Bock et al., 2005; Lerch et al., 2011). Parameters used in the anatomical MRI scans: T2- weighted 3D fast spin-echo sequence, with a cylindrical acquisition of k-space, and with a TR of 350 ms, and TEs of 12 ms per echo for six echoes, two averages, field-of-view of 20 × 20 × 25 mm^3^ and matrix size = 504 × 504 × 630 giving an image with 0.040 mm isotropic voxels (Nieman et al., 2005). The current scan time required for this sequence is ~14 h. To visualize and compare any differences in the mouse brains, the images from all brains were linearly (6 parameter followed by a 12 parameter) and non-linearly registered together, and then averaged together to create a population atlas representing the anatomy of the study sample. All registrations were performed with a combination of mni_autoreg tools (Collins et al., 1994) and advanced normalization tools (ANTs; Avants et al., 2008, 2011). The result of this registration is to have all scans deformed into alignment with each other in an unbiased fashion. This allows for the analysis of the deformations, and model how they relate to genotype (Nieman et al., 2006; Lerch et al., 2008). The Jacobian determinants of the deformation fields therefore can be used as measures of volume at each voxel. Significant volume changes were then calculated in two ways: (1) on a region basis; and (2) voxel-wise. Regional volumes are calculated by warping a pre-existing classified MRI atlas onto the population atlas. This atlas is a combination of three different atlases, comprising 159 different structures including, but not limited to, the cortical lobes, large white matter structures (i.e., corpus callosum), ventricles, cerebellum, brain stem and olfactory bulbs (Dorr et al., 2008; Ullmann et al., 2013; Steadman et al., 2014). Using the inverse transformations from the registration process, allows the labeling of the original pre-registration images in order to calculate the volumes of each individual brain region. Significant differences were determined between groups for both the 166 different regions and voxel-wise throughout the brain. Multiple comparisons in this study were controlled for using the False Discovery Rate (Genovese et al., 2002).

## Results

### Conditional Deletion of *Chd7* from the mes/r1 Region

**Figure 1:**
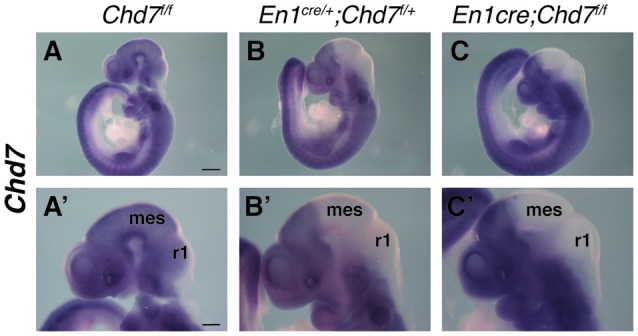
Efficient recombination of the *Chd7* conditional allele in the embryonic mid-hindbrain (mes/r1) region by *En1*^*cre*^. **(A–C)** *In situ* hybridization for *Chd7* transcripts in E9.5 *Chd7*^*f*/*f*^ **(A,A′)**, *En1*^*cre*/+^; *Chd7*^*f*/+^ **(B,B′)** and *En1*^*cre*/+^; *Chd7*^*f*/*f*^ **(C,C′)** embryos. *Chd7* expression in the mes/r1 region is reduced in *En1*^*cre*/+^; *Chd7*^*f*/+^ **(B,B′)** and lost in the *En1*^*cre*/+^; *Chd7*^*f*/*f*^ **(C,C′)**. Scale bars are 250 μm **(A–C)** and 500 μm **(A′–C′)**. **(A′–C′)** show magnified views of the corresponding images in **(A–C)**. mes = mesencephalon, r1 = rhombomere 1.

The *En1*^*cre*/+^ line has been used previously to efficiently recombine conditional alleles resulting in the loss of gene expression in most cells in the mes/r1 region by the 10 somite stage (~E8.75) (Chi et al., 2003; Basson et al., 2008). We therefore used this line to simultaneously inactivate both *Chd7* conditional alleles in the mes/r1 region in *En1*^*cre*/+^;*Chd7*^*f*/*f*^ embryos. *In situ* hybridization to detect transcripts containing *Chd7* exon 3 confirmed the ubiquitous embryonic expression of *Chd7* at E9.5 (Figure 1A), including the mes/r1 region (Figure 1A′; Randall et al., 2009). Efficient mes/r1-specific deletion of exon 3 was clearly evident in *En1*^*cre*/+^;*Chd7*^*f*/*f*^ embryos (Figures 1C,C′). Interestingly, *Chd7* expression was also clearly reduced in conditional heterozygous *En1*^*cre*/+^;*Chd7*^*f*/+^ embryos (Figures 1B,B′). *En1*^*cre*/+^, *Chd7*^*f*/*f*^ and *En1*^*cre*/+^;*Chd7*^*f*/+^ animals were born at Mendelian ratios and showed no signs of abnormalities. Although slightly fewer than expected *En1*^*cre*/+^;*Chd7*^*f*/*f*^ animals were obtained, this effect was not statistically significant (Table 1).

**Table 1:** Overall frequency of mice of each genotype from *En1*^*cre*/+^; *Chd7*^*f*/+^ × *Chd7*^*f*/*f*^ crosses at P11.

	*Chd7*^*f*/*f*^	*Chd7*^*f*/+^	*En1*^*cre*/+^; *Chd7*^*f*/+^	*En1*^*cre*/+^; *Chd7*^*f*/*f*^
Number born	10	11	13	8
Percentage spread	23.81%	26.19%	30.95%	19.05%
Expected percentage	25%	25%	25%	25%

*X^2^ = 0.40010644, indicating that there was no significant variation from expected birth frequencies. Mice were ear clipped at P11 and genotyped immediately, no postnatal mortality was noted between P0 and adulthood*.

### Structural Brain Abnormalities in *En1*^*cre*/+^; *Chd7*^*f*/*f*^ Animals

**Figure 2:**
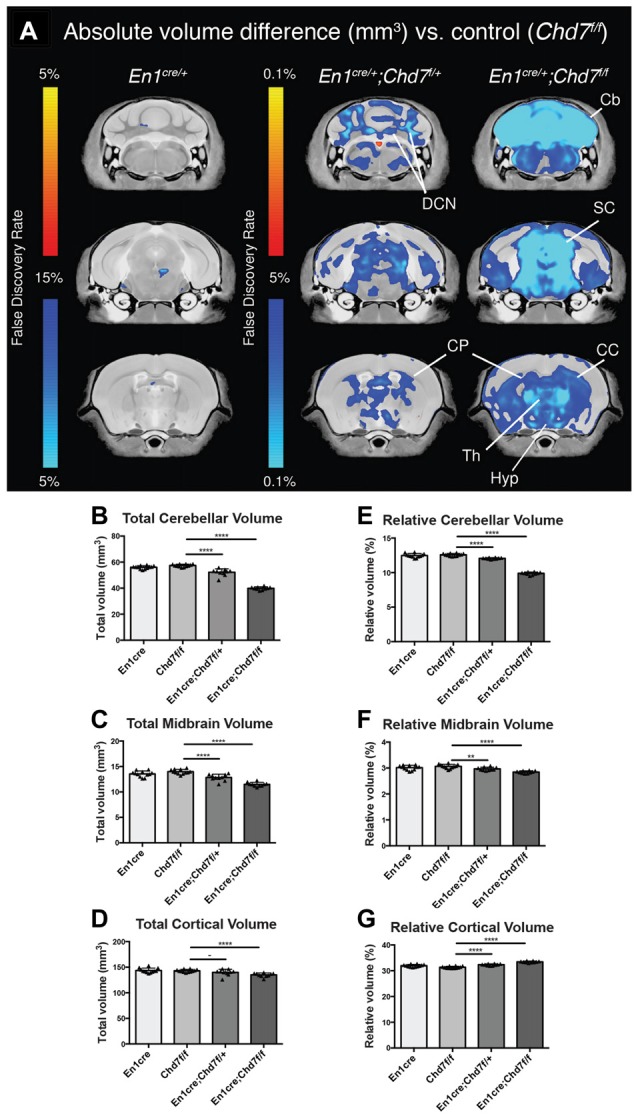
Cerebellar and midbrain hypoplasia in both heterozygous and homozygous mes/r1-specific *Chd7* conditional mutants. **(A)** Voxel-wise comparisons of high resolution 7T structural MRI coronal images of *En1*^*cre*/+^ controls (*n* = 12), *En1*^*cre*/+^; *Chd7*^*f*/+^ (*n* = 10) and *En1*^*cre*/+^; *Chd7*^*f*/*f*^ (*n* = 10) adult (P80–100) mouse brains, compared to *Chd7*^*f*/*f*^ controls (*n* = 13). Absolute volumetric differences compared to the *En1*^*cre*/+^ control are colored according to the FDR scales **(B–D)**. Areas that were larger are shown in red-yellow and areas that were smaller in dark-light blue. Absolute volumes (mm^3^) of cerebellum, midbrain and cortex plotted for *Chd7*^*f*/*f*^, *En1*^*cre*/+^, *En1*^*cre*/+^; *Chd7*^*f*/+^ and *En1*^*cre*/+^; *Chd7*^*f*/*f*^ **(E–G)** Relative volumes plotted as percentage of total brain volume. **p < 0.01 ****p < 0.005, ^−^*p* > 0.05, unpaired two-sample student’s *T*-test. Cb, cerebellum; DCN, Deep cerebellar nuclei; SC, superior colliculus; CP, caudate/putamen or Striatum; Th, Thalamus; Hyp, Hypothalamus; CC, corpus callosum.

To determine the consequences of mes/r1-specific *Chd7* deletion on brain development, brains were collected from adult animals and examined by structural MRI. We first confirmed that *En1*^*cre*/+^ animals, where one copy of the *En1* gene had been inactivated by Cre insertion and therefore heterozygous for *En1* (Kimmel et al., 2000), showed no significant structural brain anomalies, compared to *Chd7*^*f*/*f*^ controls (Figure 2A). A comparison of absolute volumes of 166 different brain regions in homozygous conditional *En1*^*cre*/+^, *Chd7*^*f*/*f*^ mutants with *Chd7*^*f*/*f*^ controls, we identified striking hypoplasia of the cerebellum and midbrain (Figures 2A–C). Intriguingly, when looking at absolute volumes, the automated MRI analysis (Supplementary Table S1) also identified other brain regions outside the mid-hindbrain region that were hypoplastic in these mutants (Figure 2A). These include the thalamus, hypothalamus and corpus callosum (Figure 2A).

Cerebellar and midbrain sizes, relative to total brain size were significantly reduced by 30.5% and 17.9%, respectively, whilst relative cortical volumes were increased by 6.7%, likely due to the sizeable decrease in cerebellar and midbrain volume (Figures 2E–G). We also found significantly reduced cerebellar (9%) and midbrain (8%) volumes in heterozygous *En1*^*cre*/+^;*Chd7*^*f*/+^ animals (Figure 2), suggesting that the reduction in *Chd7* expression in the mes/r1 in these embryos (Figures 1B,B′), had significant effects on brain development and that heterozygous *Chd7* expression during cerebellar development can be sufficient to cause mild cerebellar hypoplasia.

### *En1*^*cre*/+^; *Chd7*^*f*/*f*^ Animals Show Cerebellar Vermis Aplasia and Hypoplasia of the Hemispheres

**Figure 3:**
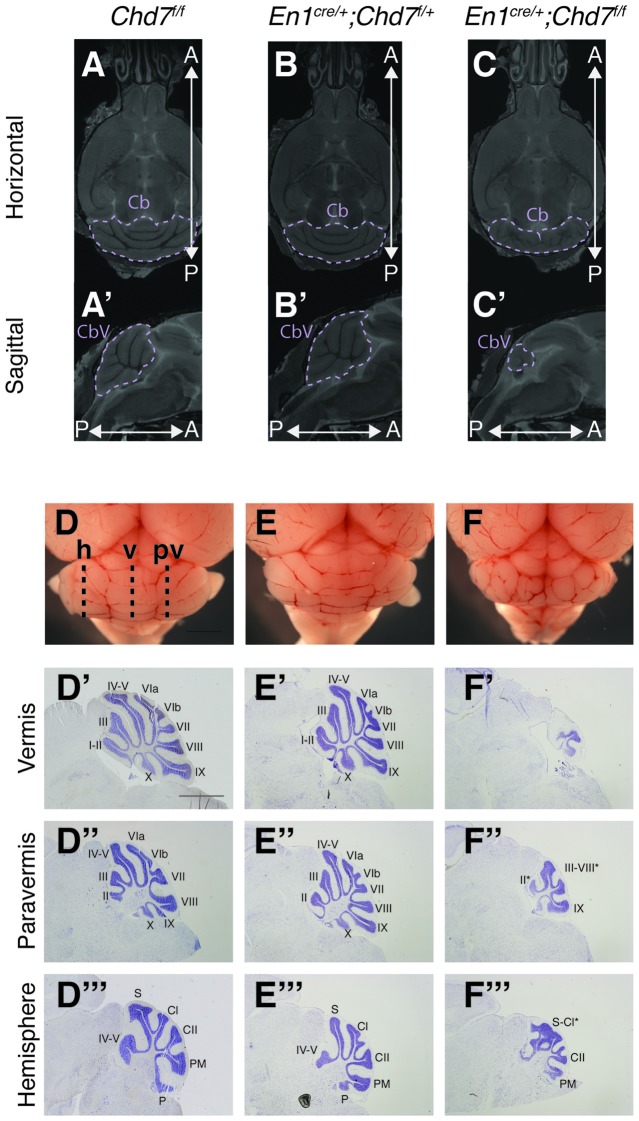
Cerebellar hypoplasia and abnormal foliation in cerebellar vermis and hemispheres of homozygous mes/r1-specific *Chd7* conditional mutants. **(A–C′)** Coronal **(A–C)** and sagittal **(A′–C′)** views of high resolution MRI images showing the cerebellum (Cb) and cerebellar vermis (CbV) of *Chd7*^*f*/*f*^ control, *En1*^*cre*/+^; *Chd7*^*f*/+^ and *En1*^*cre*/+^; *Chd7*^*f*/*f*^ adult mice. Anterior-posterior (A-P) directions are indicated. The MRI slices shown are after a linear registration and the same slices are shown in each figure. **(D–F)** Wholemount images of cerebella at P21, anterior to the top. The approximate positions of histological sections shown in **(D′–F″′)** are indicated as: h, hemisphere, v, vermis, p, paravermis. **(D′–F″′)** Cresyl violet-stained sagittal sections through the cerebellar vermis **(D′–F′)**, paravermis **(D″–F″)** and hemisphere **(D″′–F″′)** at P21, anterior to the left. Cerebellar lobules are labeled with Roman numerals according to Inouye and Oda (1980). Unlabeled lobules and asterisks in **(F′,F″,F″′)** indicate lobules with unknown identity due to highly disturbed foliation patterns. Scale bars are 1 mm **(D–F)** and 2 mm **(D′–F″′)**.

To further characterize the nature of the cerebellar hypoplasia in the *Chd7* conditional mutants, cerebellar structure was visualized in both horizontal and sagittal slices on the MRI images. Compared to *Chd7*^*f*/*f*^ and *En1*^*cre*/+^;*Chd7*^*f*/+^ animals, prominent cerebellar hypoplasia was evident in horizontal images of *En1*^*cre*/+^;*Chd7*^*f*/*f*^ brains (Figures 3A–C). and cerebellar vermis tissue was nearly absent in sagittal slices (compare Figure 3C′ with Figures 3A′,B′). The cerebellar hemispheres in these homozygous mutants were also hypoplastic and cerebellar foliation was highly irregular (Figure 3C).

Freshly collected, wholemount images taken of P21 cerebella confirmed the striking hypoplasia of the cerebellar vermis, as well as the abnormal cerebellar foliation in the hypoplastic hemispheres in *En1*^*cre*/+^;*Chd7*^*f*/*f*^ animals (Figures 3D–F). Histological sections taken at different medio-lateral positions along the cerebellum showed the near-absence of cerebellar tissue at the midline in *En1*^*cre*/+^;*Chd7*^*f*/*f*^ animals (Figure 3F′), and mild vermis hypoplasia in *En1*^*cre*/+^;*Chd7*^*f*/+^ animals (Figure 3E′) compared to controls (Figure 3D′). Sections through the paravermis revealed striking hypoplasia in the homozygous mutants compared to the other genotypes (Figures 3D″–F″). Hypoplasia of the cerebellar hemispheres and abnormal foliation was clearly evident in *En1*^*cre*/+^;*Chd7*^*f*/*f*^ mutants (compare Figure 3F″′ with Figures 3D″′,E″′).

### Midbrain Abnormalities in *En1*^*cre*/+^; *Chd7*^*f*/*f*^ Animals

**Figure 4:**
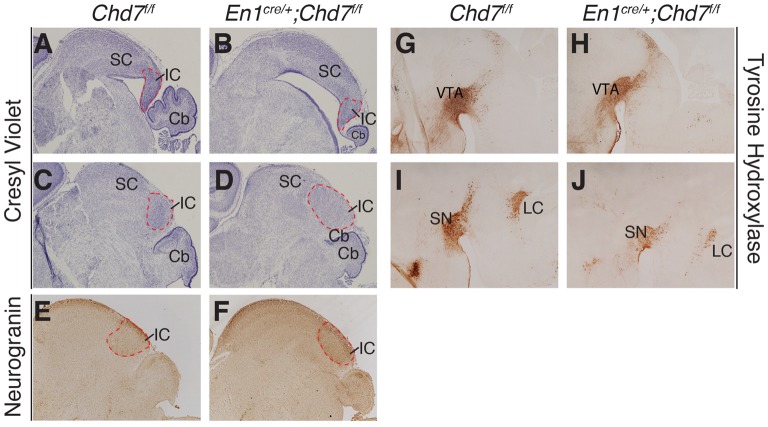
Abnormally-shaped midbrain but no loss of midbrain structures in mes/r1-specific *Chd7* conditional mutants. **(A–D)** Cresyl violet-stained mid-sagittal sections **(A,B)** and lateral sections **(C,D)** through newborn (P0) brains, with anterior to the left. The superior colliculus (SC) and inferior colliculus (IC) are labeled and the extent of the latter outlined by a broken red line. Note the striking cerebellar (Cb) hypoplasia and abnormally-shaped midbrain, but presence of both SC and IC in the conditional mutants. **(E,F)** Neurogranin immunostaining to visualize the IC in lateral sections. **(G–J)** Tyrosine hydroxylase immunostaining to visualize the ventral tegmental area (VTA) in medial sections **(G,H)** and substantia nigra (SN) in more lateral sections **(I,J)**. Images are representative of *n* = 5 **(A–D)**, *n* = 2 **(E,F)** and *n* = 4 **(G–J)** brains.

To determine whether midbrain hypoplasia in the conditional mutants (Figures 2C,F) is associated with the loss of specific midbrain structures, we examined sections from newborn animals when individual midbrain structures are easily identified. This analysis confirmed the striking cerebellar vermis hypoplasia in the homozygous, conditional mutants and revealed an abnormally-shaped midbrain (compare Figure 4A with Figure 4B). Despite these structural changes, both anterior (superior colliculus, SC) and posterior (inferior colliculus, IC) midbrain structures were present in the conditional mutants, in both medial (Figures 4A,B) and lateral (Figures 4C,D) sections. The identity of the IC was confirmed by Neurogranin immunostaining, which again confirmed the abnormally-shaped IC (Figure 4F), compared to controls (Figure 4E). Tyrosine hydroxylase immunostaining was used to visualize ventral brain structures in the diencephalon, mid- and hindbrain. In medial sections, the ventral tegmental area (VTA) was clearly present in the conditional mutants (Figures 4G,H). Immunostaining of more lateral sections (Figures 4I,J) identified the substantia nigra (SN) and the locus coeruleus (LC). Although both these structures could be identified in conditional mutants, the TH staining intensity was reduced in both (*n* = 3 mutants, compared to littermate controls), suggesting that the development of cells in these more lateral midbrain areas is affected by *Chd7* deletion from the mes/r1 region.

### *Fgf8* Gene Expression and Morphological Changes in the mes/r1 Region of Conditional *Chd7* Mutant Embryos

**Figure 5:**
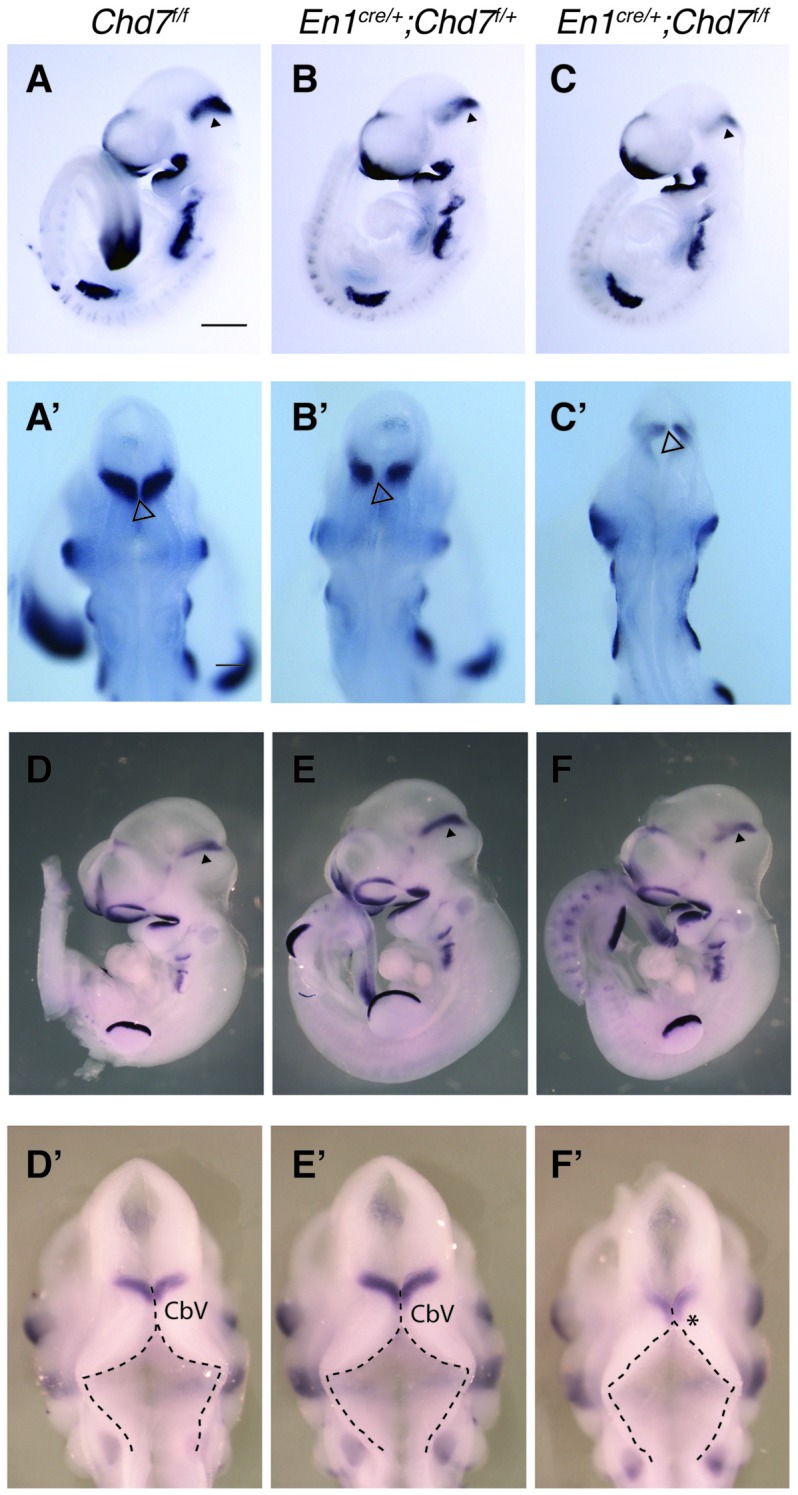
Reduced *Fgf8* expression and abnormal dorsal r1 morphology in mes/r1-specific *Chd7* conditional mutants. **(A–C)** *In situ* hybridization for *Fgf8* in E9.5 embryos of the indicated genotypes. Anterior is to the left and *Fgf8* expression in the isthmus organizer (IsO) is indicated by an arrowhead. **(A′–C′)** Dorsal views of the embryos in **(A–C)** with an open arrowhead indicating the midline roof plate at the IsO. Note the reduced *Fgf8* expression in the mutants, compared to controls. **(D–F)** *In situ* hybridization for *Fgf8* in E10.5 embryos of the indicated genotypes. Anterior is to the left and *Fgf8* expression in the IsO is indicated by an arrowhead. **(D′–F′)** Dorsal views of the embryos in **(D–F)**. The roof plate is outlined with broken lines and the approximate location of cerebellar vermis (CbV) progenitors are shown, according to Sgaier et al. (2005). Note the expanded roof plate in anterior r1 (arrow) at the expense of the CbV domain (asterisk) in homozygous conditional mutants **(F′)**, similar to other mutants with reduced fibroblast growth factor (FGF) signaling in the mes/r1 region (Basson et al., 2008). Scale bars are 500 μm. Images are representative of *n* = 3 **(A,C)**, *n* = 4 **(B)** and *n* = 2 **(D–F)** embryos.

Given the phenotypic similarities between *En1*^*cre*/+^;*Chd7*^*f*/*f*^ mutants and mutants with reduced FGF signaling (Chi et al., 2003; Basson et al., 2008), we predicted that *Fgf8* expression and signaling would be reduced in the mid-hindbrain region. Indeed, *Fgf8* expression was slightly reduced in heterozygous *En1*^*cre*/+^;*Chd7*^*f*/+^ mutants at E9.5 compared to *Chd7*^*f*/*f*^ controls (Figures 5A,B), and reduced even further in homozygous *En1*^*cre*/+^;*Chd7*^*f*/*f*^ embryos (Figure 5C). The *Fgf8* expression pattern observed from a dorsal view, showed evidence for a slightly expanded roof plate at the midline of the IsO in both the heterozygous and homozygous mutants (Figures 5A′–C′), characteristic of embryos with reduced FGF signaling at the IsO (Basson et al., 2008).

An examination of *Fgf8* expression in embryos a day later (E10.5), still showed reduced *Fgf8* expression in the IsO of homozygous mutants (compare Figure 5F with Figure 5D), whilst heterozygous mutants appeared to have normal levels of *Fgf8* expression (compare Figure 5E with Figure 5D), although subtle changes in *Fgf8* expression cannot be ruled out based on *in situ* hybridization experiments. Furthermore, viewing the dorsal aspect of r1 in these embryos, revealed a morphology typical of embryos with reduced FGF expression in homozygous mutants (Figure 5F′), compared to heterozygous and control embryos (Figures 5D′,E′). These embryos were characterized by an expanded roof plate in anterior r1, apparently at the expense of the cerebellar vermis progenitor domain, as previously reported for embryos with reduced FGF signaling in mes/r1 (Basson et al., 2008). In addition to having normal levels of *Fgf8* expression, *Chd7* heterozygous embryos also had a normal morphology, consistent with the very mild vermis hypoplasia displayed by these mutants (Figures 3E,E′).

## Discussion

The analysis of mes/r1-specific homozygous *Chd7* conditional mutants reported here provide incontrovertible support for our previous studies reporting two separate roles for *Chd7* in cerebellar development. The present study follows from our observation of mildly reduced *Fgf8* expression in *Chd7*^+/−^ embryos (Yu et al., 2013), that was found to be insufficient to fully phenocopy the striking cerebellar vermis hypoplasia observed in mutants with stronger reductions in FGF signaling. Although *Fgf8* expression was strongly reduced in *Chd7*^−/−^ embryos, the effect of this reduction in *Fgf8* expression on cerebellar vermis development could not be studied due to the embryonic lethality of these embryos (Yu et al., 2013). We report here that homozygous deletion of *Chd7* from the embryonic mes/r1 region results in vermis hypoplasia of similar severity, underpinned by strong reduction in *Fgf8* expression.

We also observed hypoplasia and abnormal foliation of cerebellar hemispheres in *En1*^*cre*/+^;*Chd7*^*f*/*f*^ mutants, consistent with our recent study that identified a role for *Chd7* in the proliferation and survival of granule neuron progenitors in the vermis and hemispheres (Whittaker et al., 2017). The striking phenotypes observed in the *En1*^*cre*/+^;*Chd7*^*f*/*f*^ mutants therefore represent a combination of two temporally distinct functions of *Chd7* during cerebellar development: (1) *Chd7* deletion from the early-mid-hindbrain region results in reduced *Fgf8* expression and signaling, leading to failure of vermis progenitors to be expanded or maintained, expansion of the roof plate and cerebellar vermis hypoplasia/aplasia; and (2) the absence of *Chd7* from granule neuron progenitors then affects the perinatal growth of the cerebellum resulting in additional hypoplasia of the hemispheres and abnormal cerebellar foliation.

Our observation of mild cerebellar hypoplasia in heterozygous *En1*^*cre*/+^;*Chd7*^*f*/+^ mutants suggest that the loss of one copy of *Chd7* is sufficient to cause a cerebellar phenotype, consistent with the presence of cerebellar vermis hypoplasia in 35% of patients with CHARGE syndrome who are haploinsufficient for *CHD7* (Yu et al., 2013).

Although the present study further supports a strong link between *Chd7* and *Fgf8* regulation in the mes/r1 region, it is curious to note that neither the conditional heterozygous mutants described here, nor the *Chd7*^+/−^ mutants reported previously, present with the loss of anterior vermis folia and loss of the IC, a phenotype typically associated with animals with reduced FGF signaling in the mes/r1 region (Xu et al., 2000; Basson et al., 2008). We propose two possible explanations for this observation: (1) the reduction in FGF signaling in *Chd7* heterozygous embryos is not sufficient to cause this phenotype; and (2) the cerebellar phenotype in *Chd7* mutants and CHARGE syndrome is complex and not solely due to reduced FGF signaling. Further experiments will be required to distinguish between these possibilities.

The observation that multiple brain regions outside the mid-hindbrain were also hypoplastic in *En1*^*cre*/+^;*Chd7*^*f*/*f*^ mutants, suggest that the striking changes in mid-hindbrain growth may impact indirectly on other brain regions. These findings may have important implications for understanding the neuroanatomical basis of complex human syndromes associated with cerebellar hypoplasia and developmental mechanisms that coordinate brain growth.

In conclusion, we show here that the homozygous, conditional deletion of *Chd7* from the early mes/r1 region results in reduced *Fgf8* expression in the IsO and anatomical phenotypes strongly linked to reduced FGF signaling, namely expansion of the roof plate and severe cerebellar vermis hypoplasia. Unlike other conditional mutants with specific disruptions of FGF signaling, these *Chd7* mutants also exhibited hypoplasia and abnormal foliation of the cerebellar hemispheres, consistent with the essential role for *Chd7* in regulating cerebellar granule neuron development during late embryonic and early postnatal development.

## Author Contributions

APAD processed brains for MRI and analyzed the data together with JE who performed the MRI and analysis with JPL. TY initiated the study and APAD and TY performed ISH and histology experiments. KLHR and CG generated and phenotyped cohorts of animals. MAB, CR-A and CF were responsible for project planning, supervision, data analysis and interpretation. MAB and APAD wrote the manuscript with input from all authors.

## Conflict of Interest Statement

The authors declare that the research was conducted in the absence of any commercial or financial relationships that could be construed as a potential conflict of interest.
